# Spontaneous Intracerebral Hemorrhage Due to Delta Storage Pool Disease in a Patient on a Serotonin-Norepinephrine Reuptake Inhibitor

**DOI:** 10.3389/fneur.2019.01257

**Published:** 2019-11-26

**Authors:** Annette Leibetseder, Judith Wagner, Josef Tomasits, Hans-Peter Haring, Markus Hutterer, Johannes Trenkler, Tim J. von Oertzen

**Affiliations:** ^1^Department of Neurology 1, Kepler University Hospital, Medical Faculty of the Johannes Kepler University Linz, Linz, Austria; ^2^Institute for Clinical Pathology, Kepler University Hospital, Medical Faculty of the Johannes Kepler University Linz, Linz, Austria; ^3^Institute of Neuroradiology, Kepler University Hospital, Medical Faculty of the Johannes Kepler University Linz, Linz, Austria

**Keywords:** intracerebral hemorrhage, stroke, SSRI, SNRI, thrombocytopathy, delta storage pool disease

## Abstract

We report a case of spontaneous intracerebral hemorrhage (sICH) due to delta storage pool disease in a 60-year-old female on a serotonin-norepinephrine reuptake inhibitor (SNRI). Increased susceptibility to SNRI-effects on hemostasis was due to a genetic disposition mediated by a polymorphism of the SLC6A4 gene coding for the human serotonin transporter (SERT). Pathophysiological and clinical implications of these findings are discussed.

## Background

Spontaneous intracerebral hemorrhage (sICH) is most often caused by hypertension, cerebral amyloid angiopathy, vascular malformation, and inherited or acquired coagulopathies (1). Platelets are an essential component of primary hemostasis. Their activation is initiated and maintained by small molecules released from dense granules contained within the platelets (2). Serotonin is among the molecules stored in platelet granules. Upon release, it promotes platelet aggregation via the 5-HT_2A_ receptor. Number or content of dense granules is reduced in delta storage pool disease, a rare and etiologically heterogeneous platelet disorder (3).

Uptake of serotonin into the platelet cytosol is mediated via the serotonin transporter (SERT), which is identical to the one found in neurons. SERT is coded by the SLC6A4 gene on chromosome 17 (4, 5). Serotonergic antidepressants such as selective serotonin reuptake inhibitors (SSRIs) and serotonin-norepinephrine reuptake inhibitors (SNRI) are known to reduce platelet serotonin content (6). Use of SSRIs is also associated with an increased risk for sICH as recently shown in a population-based study (7). The extent of the SSRI effect on platelet function is related to an insertion/deletion polymorphism in the promoter region (5-HTTLPR; serotonin-transporter-linked polymorphic region) of the SLC6A4 gene coding for the human SERT. The 5-HTTLPR gene has a short (S) and a long (L) allele, the S variant being associated with decreased transcription. A higher sensitivity to serotonergic antidepressants—and hence a higher risk of hemorrhage—may be seen in the short gene (SS) polymorphism (8).

## Case Presentation

**Figure 1:**
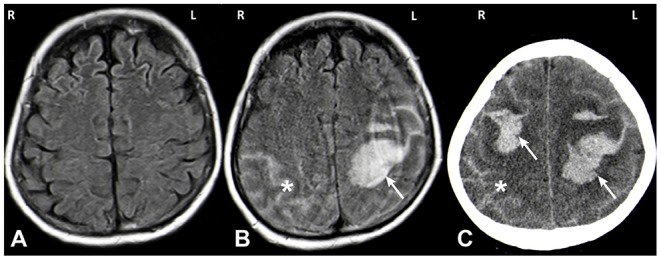
MRI **(A,B)** and CT **(C)** images. MRI imaging on day of admission after a first seizure was unremarkable (**A**: axial FLAIR). MRI on day 5 showed a spontaneous left hemispheric ICH with a subarachnoid component (SAB) (**B**: axial FLAIR; white arrow: ICH, asterisk: SAB). One day later the patient deteriorated again and CT imaging showed a right sided sICH and edema of the left hemisphere (**C**: axial CT; white arrow: ICH, asterisk: SAB).

We present the case of a 66-year old female who was admitted to our hospital due to a first generalized tonic-clonic seizure. The patient did not report any symptoms suggestive of an epileptic aura. She denied any other focal neurological symptoms, nausea or headaches. The remaining neurological examination was unremarkable except for disorientation as to the situation. Initial investigations including cerebral magnetic resonance imaging (MRI; Figure 1A) and cerebrospinal fluid (CSF) analysis were negative. Blood tests showed no signs for infection or metabolic abnormalities.

The patient had a history of recurrent episodes of major depression and was treated with the SNRI venlafaxine 150 mg per day. She had started venlafaxine 14 years before the current event, taking doses of 100–150 mg per day (an increase to 225 mg had been suggested earlier—this change had apparently not been implemented by the patient). The combined plasma level of venlafaxine and its active metabolite O-desmethyl venlafaxine was elevated (541 ng/ml, range 100–400 ng/ml). Due to ongoing major depression, the venlafaxine dose was increased to 225 mg/day on the day after hospitalization. At the time of admission, the patient took amisulpride, prothipendyl, hydroxyzine, and zolpidem tartrate in addition to venlafaxine, but no other antidepressant. Within the period of 14 years documented in the patient file, she had not been medicated with another SNRI or SSRI. The only other antidepressant medication tried apart from venlafaxine was trazodone (maximal dose 250 mg/day).

Five days after hospitalization, the patient suffered a spontaneous left hemispheric intracranial hemorrhage with a large intraparenchymal and a small subarachnoidal component (Figure 1B). On the subsequent day, a second bleed occurred on the contralateral side (Figure 1C). Conventional angiography displayed local rarefication of cerebral vessels, most likely secondary to the hemorrhage. Vasculitis, reversible vasoconstriction syndrome and vascular malformations were ruled out with this method. Magnetic resonance (MR) imaging and MR angiography did not show any signs of cerebral amyloid angiopathy, cerebral venous thrombosis, brain metastases, or other suspicious lesions. Cerebrospinal fluid diagnostics exhibited no abnormalities.

On examination of the coagulation system, a disorder of platelet aggregation was diagnosed. Immunofluorescence microscopy revealed a decrease of the granule markers Lamp 1/2 and CD63, compatible with delta storage pool disease. We assumed a drug-induced pathogenesis due to venlafaxine and replaced it with mirtazapine. Two weeks after discontinuing venlafaxine, the platelet function tests yielded normal results. SERT-promoter sequencing in our patient revealed a heterozygote genotype (SL).

## Discussion

In conclusion, we diagnosed an acquired form of delta-storage pool deficiency induced by venlafaxine in a patient with a genetic predisposition due to a heterozygote genotype (SL) of the SLC6A4 gene coding for the platelet SERT. Whereas, patients homozygous for the LL genotype have not displayed an increased bleeding time after SSRI treatment, those with a heterozygote (SL) or homozygote (SS) genotype have (8).

A dose-dependent correlation between antidepressant intake and platelet dysfunction has been found for the selective noradrenaline reuptake inhibitor desipramine. Reduction of platelet serotonin content was proportional to the steady state plasma level of the drug (6). These findings increase the likelihood of our patient's hemorrhage being due to the increased venlafaxine dose she had received.

To our knowledge, there is no other case on delta storage pool disease and sICH reported in adults on SNRI. However, it is difficult to estimate how often patients with sICH while on this medication are actually investigated for platelet storage disease. We suggest investigating for impaired platelet aggregation and/or SLC6A4-genotyping in patients with spontaneous intracranial hemorrhage or other serious bleeding while on serotonergic antidepressants. Considering all risks and benefits, a switch to a non-serotonergic antidepressant may be considered in an SS- or SL-genotype.

Pre-therapeutic screening for susceptibility to adverse effects would require SLC6A4 genotyping as platelet function tests are likely to be normal in this context. With an odds ratio for a hemorrhage of about 3 in a patient on SSRI (and potentially even lower for patients on SNRIs), determination of the SERT polymorphism in all patients prior to starting with an SSRI or SNRI will not be cost-effective (9). However, standard screening for thrombocytopathies in patients already on SSRI or SNRI may be advisable (10). Alternatively, immunocytochemical assays might be helpful in detecting abnormal serotonin levels in platelets of patients on serotonergic antidepressants (11).

Intensive care physicians, stroke specialists as well as psychiatrists should be aware of severe bleeding—including sICH—as a potential side-effect in patients on SNRIs.

The cause of the epileptic seizure in this patient could not be fully elucidated. MRI and MR angiography on the day of admission did not reveal any acute alterations, particularly no signs of reversible vasoconstriction syndrome or cerebral venous thrombosis. Neither did a CSF count, a full blood and serum analysis or a toxicology screen. Seizures provoked by therapeutic doses of venlafaxine have been reported (12). However, our patient had been on the same dose of venlafaxine for many years and never suffered from seizures before. Nor was there any other change of medication before the seizure occurred. Epidemiological studies have shown an increased risk of subsequent cerebrovascular disease—particularly of hemorrhagic stroke—in patients with late-onset seizures (13). The causal mechanism of this correlation has not yet been fully elucidated. In our patient though, the predisposition for hemorrhagic stroke—namely the increase in venlafaxine dose leading to thrombocytic dysfunction—only occurred after hospital admission. Thus, the seizure can hardly be defined as a pre-stroke seizure. Finally, a bidirectional relationship between depression and epilepsy, possibly due to shared pathophysiological mechanisms, has been described (14). Hence, we diagnosed a late-onset seizure in a patient belonging to an epidemiological risk group and with a potentially lowered seizure threshold due to venlafaxine treatment.

## Ethics Statement

All subjects gave written informed consent in accordance with the Declaration of Helsinki. The patient gave written informed consent for genetic analysis. The study was exempt from ethical approval procedures.

## Author Contributions

AL, JW, and TO conceived the idea of this work. AL and JW collected the data. AL, JW, TO, JT, H-PH, MH, and JTr helped with data analysis and interpretation, drafting of the article, and critical revision of the article.

### Conflict of Interest

The authors declare that the research was conducted in the absence of any commercial or financial relationships that could be construed as a potential conflict of interest.
